# 3D active workspace of human hand anatomical model

**DOI:** 10.1186/1475-925X-6-15

**Published:** 2007-05-02

**Authors:** Doina Dragulescu, Véronique Perdereau, Michel Drouin, Loredana Ungureanu, Karoly Menyhardt

**Affiliations:** 1 Department of Mechanics and Vibrations, Politehnica University of Timisoara, 1 M. Viteazul Boulevard, 300222 Timisoara, Romania; 2 LISIF laboratory, Pierre et Marie Curie University, Paris VI, 3 Rue Galilee, 94200 Ivry-Sur-Seine, France; 3 Department of Automation and Applied Informatics, Politehnica University of Timisoara, 2 Vasile Parvan Boulevard, 300223 Timisoara, Romania

## Abstract

**Background:**

If the model of the human hand is created with accuracy by respecting the type of motion provided by each articulation and the dimensions of articulated bones, it can function as the real organ providing the same motions. Unfortunately, the human hand is hard to model due to its kinematical chains submitted to motion constraints. On the other hand, if an application does not impose a fine manipulation it is not necessary to create a model as complex as the human hand is. But always the hand model has to perform a certain space of motions in imposed workspace architecture no matter what the practical application does.

**Methods:**

Based on Denavit-Hartenberg convention, we conceived the kinematical model of the human hand, having in mind the structure and the behavior of the natural model. We obtained the kinematical equations describing the motion of every fingertip with respect to the general coordinate system, placed on the wrist. For every joint variable, a range of motion was established. Dividing these joint variables to an appropriate number of intervals and connecting them, the complex surface bordering the active hand model workspace was obtained.

**Results:**

Using MATLAB 7.0, the complex surface described by fingertips, when hand articulations are all simultaneously moving, was obtained. It can be seen that any point on surface has its own coordinates smaller than the maximum length of the middle finger in static position. Therefore, a sphere having the centre in the origin of the general coordinate system and the radius which equals this length covers the represented complex surface.

**Conclusion:**

We propose a human hand model that represents a new solution compared to the existing ones. This model is capable to make special movements like power grip and dexterous manipulations. During them, the fingertips do not exceed the active workspace encapsulated by determined surfaces. The proposed kinematical model can help to choose which model joints could be eliminated in order to preserve only the motions important for a certain application. The study shows that all models, simplified or not, exhibit a pronounced similitude with the real hand motion, validated by the fingertips' computed trajectories. The results were used to design an artificial hand capable to make some of the hand's functions with a reduced set of degrees of freedom.

## Background

The human hand consists of connected parts composing kinematical chains so that hand motion is highly articulated. At the same time, many constraints among fingers and joints make the hand motion even harder to model. Various models of the human hand have been already created worldwide. Vardy proposes a 26 degrees of freedom (DoFs) hand model, based on Denavit-Hartenberg convention [1]. All fingers have the same essential structure, so the convention is applied to all fingers in the same manner. Each finger has five DoFs: one DoF corresponding to the part of carpometacarpal articulation considered as belonging to the respective finger, two DoFs corresponding to metacarpophalangeal articulation, and one DoF corresponding to every interphalangeal articulation. The thumb has a different structure: three DoFs corresponding to the carpometacarpal (CMC) articulation, two DoFs corresponding to metacarpophalangeal (MCP) articulation, and one DoF corresponding to the interphalangeal (IP) articulation. So, in the model from [1] the wrist was neglected and the palm was imagined as a seven DoF's articulation. The model is no complete because the applied constraints are merely rough approximations of the kind of restrictions in the real human hand. The global reference frame is placed on wrist, the final motions of fingertips being analyzed with respect to this frame.

Very similar with the model from [1] is the model proposed by Yasumuro in [2]. This model has the same structure, only the CMC articulation of the thumb has two DoFs. Also, the wrist is modeled as having six DoFs: three rotations and three translations. The fix coordinate system with respect to which the whole motion is analyze is placed outside of the hand's area. Yasumuro uses this model to create, from surfaces, a 3D model of the human hand and to animate it, based on a dynamic model, in a human like manner, even when a few hand parameters are available.

Also very similar with the model from [1] and used for animation, as in [2], is the model from [3]. The structure is the same as in [1] but the number of DoFs in CMC area is different: the thumb has three DoFs, the ring and little fingers have two DoFs and index and middle fingers have no motion.

Wu and Huang [4] treated the hand as a set of sub-objects, each of them being separately modeled. The skeleton of a hand was abstracted as a stick figure so that the dimension of each sub-object was reduced to its link length. Each finger is modeled as a kinematical chain with the palm as its base reference frame. The model does not consider the radiocarpal articulation (wrist). Each fingertip is the end-effector of the respective finger kinematical chain.

Based on two models, [5] and [6], a kinematical model intended to be suited for measuring and displaying fine fingertip manipulations was developed in [7]. The base coordinate system was located in the hand at the point where the thumb and the index metacarpal meet. The index finger was defined similarly to that presented in [6]. The model studies only these fingers, the three others adopting the index model.

A 27 DoFs model of the hand with some simplifying assumptions concerning thumb's motion and independency of fingers, joints and hands motion is proposed in [8]. These authors set up the groundwork for a more complete anatomically based hand model that can be fitted to and validated by human motion data.

Considering the tracking of a fingertip in space, then the volume generated by every possible point touched by this finger is called workspace of that limb [9]. The boundary of this workspace is always a surface, referred by some authors as "reach envelope"[10,11]. The complete identification of the workspace is very important to quantify the full functional potential of the joint and to study ergonomic postures and motion path trajectories [9].

Significant attention was given to determine the workspace of the normal and impaired hand. There are studies ([12,13]) regarding the limitation of joint rotation on the independence of hand rotation, but the workspace determination was altered by the number of DoFs modeled and by the numerical algorithms used. In [9] Denavit-Hurtenberg convention was used to describe the kinematical chains of the human hand and the Jacobian matrix to identify boundary surfaces. They present the workspace of a finger and visualize the progress of the wrist (modeled as a 3 DoFs system) after a surgical procedure (when the total workspace is limited) using a surface area calculation.

Kim et all deals in [14] with a three finger grasping system, which is usually used in writing, soldering, and surgery. Based on the fact that the motion of the wrist is independent from the motion of the finger, they calculated the workspace independently, for the thumb and the wrist (in flexion and extension, adduction and abduction motions). To solve the workspace for that particular grasping system, an analytical approach was used, considered reliable since bone to bone bonyratio is fixed.

The aim of the study is to obtain a human hand model, as natural as possible, representing the theoretical basis for functional hand prosthesis capable to realize various tasks in 3D environment (which is already constructed, but we are still working at the control algorithms). That is why the motion was studied by representing the active space as a complex surface (reach envelope) inside of which the hand model will surely appear during any task. The intersections between this active space and the global reference frame planes represent the fingertips trajectories. The model correctness was appreciated by comparing these trajectories to the real ones. It takes into consideration the constraints imposed by the joints specific geometry characterized by minimum and maximum angles values [15].

## Methods

The kinematical model of the human hand was conceived conformably to Denavit-Hartenberg convention [16,17], taking into account the real anatomical bones chains [18]. The highly articulated human hand was modeled, based on the anatomical structure, as a 22 DoFs bodies system linked by:

• one spherical joint representing the radiocarpal (wrist) articulation, realized by superposing three revolute orthogonal joints;

• 19 revolute joints representing fingers' articulations where, for every finger, the proximal articulation has superposed two orthogonal revolute joints and all of the others, only one revolute joint; the fingers are linked to the palm in different points and are modeled as independent kinematical chains.

These kinematical chains contain rigid bodies connected through the mentioned joints. The first body is the palm, linking together the wrist and the proximal phalange of each finger, which is the second body of each kinematical chain. The wrist allows the rotation of the hand with respect to the arm, meaning three DoFs for the hand system. Each of the four central fingers has four DoFs. The MCP joint allows two kinds of motions (two DoFs) to the proximal phalange of a finger: adduction-abduction and flexion-extension. The proximal interphalangeal (PIP) joint connects the proximal and medial phalanges and has one DoF. The distal interphalangeal (DIP) joint connects the medial and distal phalanges and has also only one DoF. The thumb has a different structure having three DoFs, one for the IP joint and two for the MCP joint, due to flexion-extension and abduction-adduction finger motions. Therefore, the thumb is able to move in opposition with other fingers.

### Kinematical model

**Figure 1:**
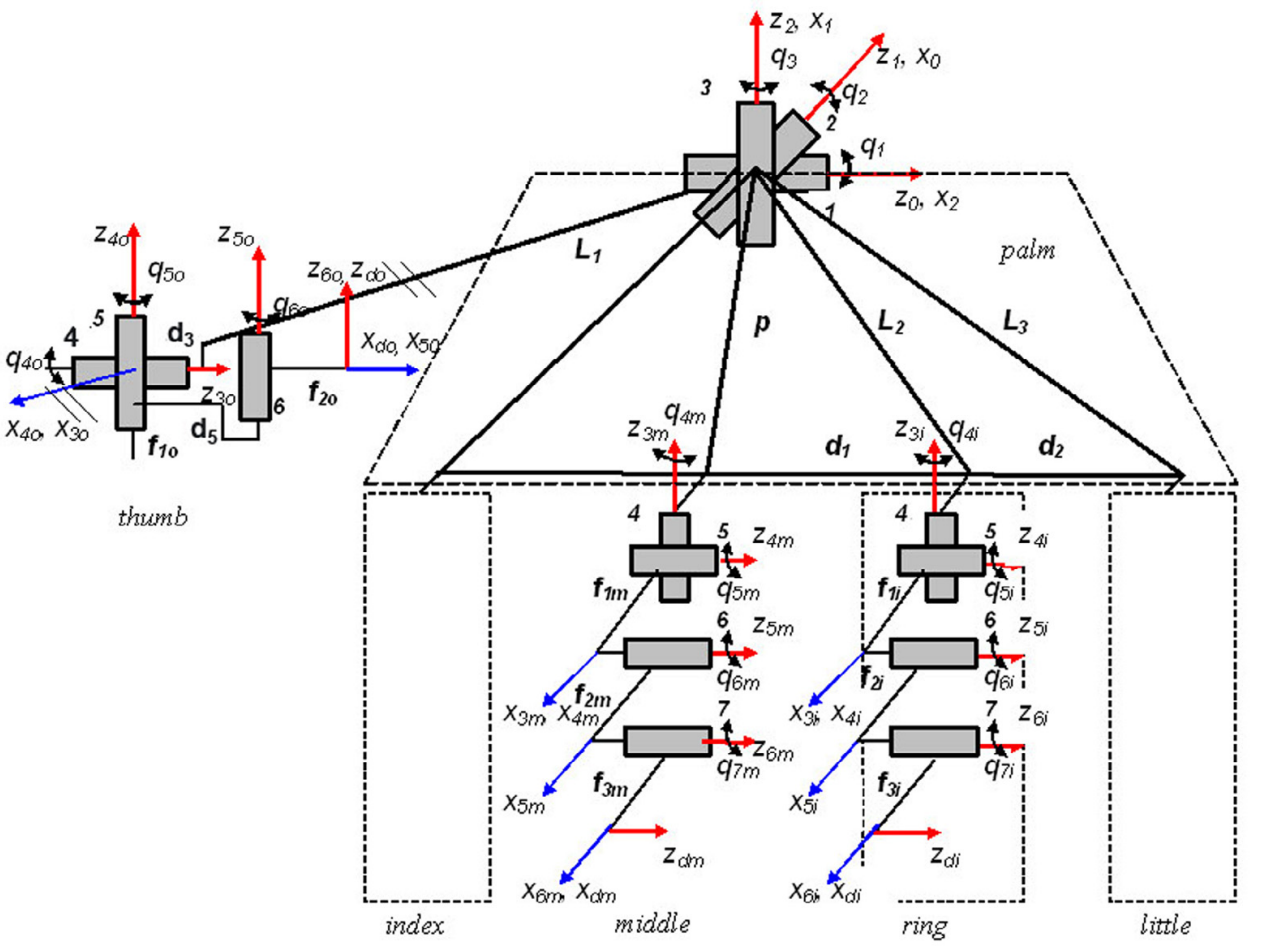
**Kinematical scheme of human hand**. The kinematical scheme of the human hand contains the palm and the phalanges as rigid bodies linked by 22 revolute joints. The reference frame *x*_*i*_*O*_*i*_*y*_*i*_*z*_*i *_is placed on the joint *i *+ 1. The joint variables are *q*_1_, *q*_2_, *q*_3_, *q*_4*k*_, *q*_5*k*_, *q*_6*k*_, *q*_7*k *_(*k *= *o*, *m*, *i*, *l*), the three first corresponding to the wrist, common articulation for all fingers. The bending angle *α*_*i *_is measured between *O*_*i*-1_*z*_*i*-1 _and *O*_*i*_*z*_*i *_axes conformably to the positive sense of the *O*_*i*_*x*_*i*_. It can be observed that, in the case of the lateral fingers (pinky, ring finger and index), the kinematical chain structure is the same as for the middle finger, the only difference being the values of distances *L*_*k *_between every origin of the reference frames attached to the proximal fingers' phalanges and the fixed global *x*_0_*O*_0_*y*_0_*z*_0 _one.

The kinematical scheme of the human hand (Figure 1 and Table 1) was realized [19,20] by considering 22 revolute joints linking together the palm and the phalanges as rigid bodies. The reference frame *x*_*i*_*O*_*i*_*y*_*i*_*z*_*i *_is placed on the joint *i+1*, as follows: *O*_*i*_*z*_*i *_corresponds to the joint *i+1 *axis, *O*_*i*_*x*_*i *_follows the link *i *length, being orthogonal to the corresponding *i *and *i+1 *joints axes. *O*_*i*_*y*_*i *_is orthogonal to the two axes before mentioned, but not important for the model. The joint variables are *q*_1_, *q*_2_, *q*_3_, *q*_4*k*_, *q*_5*k*_, *q*_6*k*_, *q*_7*k *_(*k *= *o*, *m*, *i*, *l*), the three first corresponding to the wrist, common articulation for all fingers. The bending angle *α*_*i *_is measured between *O*_*i*-1_*z*_*i*-1 _and *O*_*i*_*z*_*i *_axes conformably to the positive sense of the *O*_*i*_*x*_*i*_. It can be observed that, in the case of the lateral fingers (pinky, ring finger and index), the kinematical chain structure is the same as for the middle finger, the only difference being the values of distances *L*_*k *_between every origin of the reference frames attached to the proximal fingers' phalanges and the fixed global *x*_0_*O*_0_*y*_0_*z*_0 _one.

**Table 1:** The geometrical parameters of the proposed hand model

***Thumb***					***Other finger***				
***No***.	***θ*_*i*_**	***d*_*i*_**	***L*_*i*_**	***α*_*i*_**	***No***.	***θ*_*i*_**	***d*_*i*_**	***L*_*i*_**	***α*_*i*_**

(1)	(2)	(3)	(4)	(5)	(6)	(7)	(8)	(9)	(10)
1	*q*_*1*_	0	0	90^0^	1	*q*_1_	0	0	90^0^
2	*q*_*2*_	0	0	90^0^	2	*q*_2_	0	0	90^0^
3o	*q*_*3o*_	*d*_*3*_	*L*_*o *_= *L*_*1*_	-90^0^	Lm=PLi=p2+d12=L2Ll=p2+(d1+d2)2=L3 MathType@MTEF@5@5@+=feaafiart1ev1aaatCvAUfKttLearuWrP9MDH5MBPbIqV92AaeXatLxBI9gBaebbnrfifHhDYfgasaacH8akY=wiFfYdH8Gipec8Eeeu0xXdbba9frFj0=OqFfea0dXdd9vqai=hGuQ8kuc9pgc9s8qqaq=dirpe0xb9q8qiLsFr0=vr0=vr0dc8meaabaqaciaacaGaaeqabaqabeGadaaakeaafaqaaeWabaaabaGaemitaW0aaSbaaSqaaiabd2gaTbqabaGccqGH9aqpcqWGqbauaeaacqWGmbatdaWgaaWcbaGaemyAaKgabeaakiabg2da9maakaaabaGaemiCaa3aaWbaaSqabeaacqaIYaGmaaGccqGHRaWkcqWGKbazdaqhaaWcbaGaeGymaedabaGaeGOmaidaaaqabaGccqGH9aqpcqWGmbatdaWgaaWcbaGaeGOmaidabeaaaOqaaiabdYeamnaaBaaaleaacqWGSbaBaeqaaOGaeyypa0ZaaOaaaeaacqWGWbaCdaahaaWcbeqaaiabikdaYaaakiabgUcaRiabcIcaOiabdsgaKnaaBaaaleaacqaIXaqmaeqaaOGaey4kaSIaemizaq2aaSbaaSqaaiabikdaYaqabaGccqGGPaqkdaahaaWcbeqaaiabikdaYaaaaeqaaOGaeyypa0JaemitaW0aaSbaaSqaaiabiodaZaqabaaaaaaa@52CE@				
4o	*q*_*4o*_	0	0	90^0^					
5o	*q*_*5o*_	*d*_*5*_	*f*_*1o*_	0^0^					
6o	*q*_*6o*_	0	*f*_*2o*_	0^0^					
Lo=(p2)2+(d1+d2)2d3,d5=negatives MathType@MTEF@5@5@+=feaafiart1ev1aaatCvAUfKttLearuWrP9MDH5MBPbIqV92AaeXatLxBI9gBaebbnrfifHhDYfgasaacH8akY=wiFfYdH8Gipec8Eeeu0xXdbba9frFj0=OqFfea0dXdd9vqai=hGuQ8kuc9pgc9s8qqaq=dirpe0xb9q8qiLsFr0=vr0=vr0dc8meaabaqaciaacaGaaeqabaqabeGadaaakeaafaqabeGabaaabaGaemitaW0aaSbaaSqaaiabd+gaVbqabaGccqGH9aqpdaGcaaqaamaabmaabaWaaSaaaeaacqWGWbaCaeaacqaIYaGmaaaacaGLOaGaayzkaaWaaWbaaSqabeaacqaIYaGmaaGccqGHRaWkcqGGOaakcqWGKbazdaWgaaWcbaGaeGymaedabeaakiabgUcaRiabdsgaKnaaBaaaleaacqaIYaGmaeqaaOGaeiykaKYaaWbaaSqabeaacqaIYaGmaaaabeaaaOqaaiabdsgaKnaaBaaaleaacqaIZaWmaeqaaOGaeiilaWIaemizaq2aaSbaaSqaaiabiwda1aqabaGccqGH9aqpcqWGUbGBcqWGLbqzcqWGNbWzcqWGHbqycqWG0baDcqWGPbqAcqWG2bGDcqWGLbqzcqWGZbWCaaaaaa@5276@					3 k	*q*_*3k*_	0	*L*_*k*_	0^0^
					4k	*q*_*4k*_	0	0	-90^0^
					5k	*q*_*5k*_	0	*f*_*1k*_	0^0^
					6k	*q*_*6k*_	0	*f*_*2k*_	0^0^
					7k	*q*_*7k*_	0	*f*_*3k*_	0^0^
					*k *= *m *for the middle finger *k *= *i *for index and ring finger *k *= *l *for pinky				

The table contains the geometrical parameters for every kinematical chain of the proposed model. These were obtained based on Denavit-Hartenberg convention from Figure 1.

By considering the general expression of the matrix assuring the transfer from a reference frame to the next one:

Tii-1=[cos⁡qi−sin⁡qicos⁡αisin⁡qisin⁡αiLicos⁡qisin⁡qicos⁡qicos⁡αi−cos⁡qisin⁡αiLisin⁡qi0sin⁡αicos⁡αidi0001]

all transfer matrices were written for all fingers separately, whose phalanges functioned as open kinematical chains.

By multiplying the corresponding transfer matrices written for every finger, the kinematical equations describing the fingertip motion with respect to the general coordinate system (axes orientations and origin position of the reference frame attached to the finger distal tip), were determined as a general matrix:

Gn0=[nxoxaxpxnyoyaypynzozazpz0001]

where *n *= 6 for thumb and *n *= 7 for all other fingers. The three first columns contain direction cosines characterizing the axes orientation and the last column contains the parametrical equations of the fingertips motions. The last column elements were used to represent the active space described by the fingertips when all joints angular domains are covered between the limits established as functions of constraints.

### Modeling the constraints

The palm and fingers assembly model is constrained and so, the real hand cannot make arbitrary gestures because of linking tendons and muscles that govern the motion. Hand geometrical constraints were considered [15,21] in order to limit the finger motions because of hand anatomy and gestures correctness, as follows:

• for middle and lateral fingers:

−90°≤q1≤80°−30°≤q2≤120°−15°≤q3≤30°−10°≤q4k≤20°−15°≤q5k≤85°0°≤q6k≤90°0°≤q7k≤70°
 MathType@MTEF@5@5@+=feaafiart1ev1aaatCvAUfKttLearuWrP9MDH5MBPbIqV92AaeXatLxBI9gBaebbnrfifHhDYfgasaacH8akY=wiFfYdH8Gipec8Eeeu0xXdbba9frFj0=OqFfea0dXdd9vqai=hGuQ8kuc9pgc9s8qqaq=dirpe0xb9q8qiLsFr0=vr0=vr0dc8meaabaqaciaacaGaaeqabaqabeGadaaakeaafaqaaeGaeaaaaeaacqGHsislcqaI5aqocqaIWaamcqqGWcaScqGHKjYOcqWGXbqCdaWgaaWcbaGaeGymaedabeaakiabgsMiJkabiIda4iabicdaWiabbclaWcqaaiabgkHiTiabiodaZiabicdaWiabbclaWkabgsMiJkabdghaXnaaBaaaleaacqaIYaGmaeqaaOGaeyizImQaeGymaeJaeGOmaiJaeGimaaJaeeiSaalabaGaeyOeI0IaeGymaeJaeGynauJaeeiSaaRaeyizImQaemyCae3aaSbaaSqaaiabiodaZaqabaGccqGHKjYOcqaIZaWmcqaIWaamcqqGWcaSaeaacqGHsislcqaIXaqmcqaIWaamcqqGWcaScqGHKjYOcqWGXbqCdaWgaaWcbaGaeGinaqJaem4AaSgabeaakiabgsMiJkabikdaYiabicdaWiabbclaWcqaaiabgkHiTiabigdaXiabiwda1iabbclaWkabgsMiJkabdghaXnaaBaaaleaacqaI1aqncqWGRbWAaeqaaOGaeyizImQaeGioaGJaeGynauJaeeiSaalabaGaeGimaaJaeeiSaaRaeyizImQaemyCae3aaSbaaSqaaiabiAda2iabdUgaRbqabaGccqGHKjYOcqaI5aqocqaIWaamcqqGWcaSaeaacqaIWaamcqqGWcaScqGHKjYOcqWGXbqCdaWgaaWcbaGaeG4naCJaem4AaSgabeaakiabgsMiJkabiEda3iabicdaWiabbclaWcqaaaaaaaa@952D@

• for thumb:

−90°≤q1≤80°−30°≤q2≤120°−15°≤q3≤30°−10°≤q4o≤20°−0°≤q5o≤80°10°≤q6o≤90°
 MathType@MTEF@5@5@+=feaafiart1ev1aaatCvAUfKttLearuWrP9MDH5MBPbIqV92AaeXatLxBI9gBaebbnrfifHhDYfgasaacH8akY=wiFfYdH8Gipec8Eeeu0xXdbba9frFj0=OqFfea0dXdd9vqai=hGuQ8kuc9pgc9s8qqaq=dirpe0xb9q8qiLsFr0=vr0=vr0dc8meaabaqaciaacaGaaeqabaqabeGadaaakeaafaqaaeGaeaaaaeaacqGHsislcqaI5aqocqaIWaamcqqGWcaScqGHKjYOcqWGXbqCdaWgaaWcbaGaeGymaedabeaakiabgsMiJkabiIda4iabicdaWiabbclaWcqaaiabgkHiTiabiodaZiabicdaWiabbclaWkabgsMiJkabdghaXnaaBaaaleaacqaIYaGmaeqaaOGaeyizImQaeGymaeJaeGOmaiJaeGimaaJaeeiSaalabaGaeyOeI0IaeGymaeJaeGynauJaeeiSaaRaeyizImQaemyCae3aaSbaaSqaaiabiodaZaqabaGccqGHKjYOcqaIZaWmcqaIWaamcqqGWcaSaeaacqGHsislcqaIXaqmcqaIWaamcqqGWcaScqGHKjYOcqWGXbqCdaWgaaWcbaGaeGinaqJaem4Ba8gabeaakiabgsMiJkabikdaYiabicdaWiabbclaWcqaaiabgkHiTiabicdaWiabbclaWkabgsMiJkabdghaXnaaBaaaleaacqaI1aqncqWGVbWBaeqaaOGaeyizImQaeGioaGJaeGimaaJaeeiSaalabaGaeGymaeJaeGimaaJaeeiSaaRaeyizImQaemyCae3aaSbaaSqaaiabiAda2iabd+gaVbqabaGccqGHKjYOcqaI5aqocqaIWaamcqqGWcaSaeaaaeaaaaaaaa@8725@

By varying all joints variables in the ranges (3) and (4) defined by constraints it was possible to represent the complex surface described by the fingers' tips with respect to the global reference frame. Any configuration of the hand segments, provided by joints' independent or correlated motions, will be doubtlessly found inside the complex surface when all joints move simultaneously, or only some of them. From this reason the interfinger constraints, which also assures the natural motion of the hand, were not considered.

### Motion study

**Figure 2:**
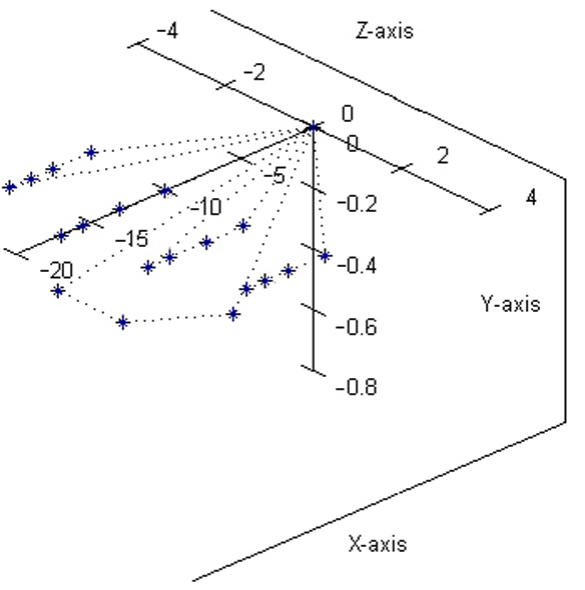
**Static posture of the human hand, as starting position for motion**. This figure represents the human hand position considered as initial for all assembly configurations. Four fingers are placed in horizontal plane *x*_0_*O*_0_*z*_0 _and the thumb is pendant in a vertical plane parallel with *x*_0_*O*_0_*y*_0_. The middle finger is placed along the *O*_0_*x*_0 _axis.

By introducing in the parametrical equations the anatomical concrete values (measured on an adult healthy hand) *d*_1 _= *d*_2 _= 2 *cm*, *d*_3 _= 0.5 *cm*, *d*_5 _= 0.2 *cm*, *p *= 10 *cm*, *f*_1*o *_= *f*_1*i *_= *f*_1*l *_= *f*_2*m *_= 2.5 *cm*, *f*_1*m *_= 3 *cm*, *f*_2*o *_= *f*_2*i *_= *f*_2*l *_= *f*_3*m *_= 1.5 *cm*, *f*_3*i *_= *f*_3*l *_= 1 *cm*, with which *L*_*o *_= 6.40 *cm*, *L*_*i *_= 10.19 *cm*, *L*_*m *_= 10 *cm*, *L*_*l *_= 10.77 *cm *were computed, it was possible to represent the human hand position considered as initial for all assembly configurations (Figure 2). It results from the figure that, four fingers are placed in horizontal plane *x*_0_*O*_0_*z*_0 _and the thumb is pendant in a vertical plane parallel with *x*_0_*O*_0_*y*_0_. The middle finger is placed along the *O*_0_*x*_0 _axis, with the distal phalange tip at the distance - (*p *+ *f*_1*m *_+ *f*_2*m *_+ *f*_3*m*_) = -17 *cm *with respect to the fixed point *O*_0_.

Every joint variable range, conformably to the constraints (3) and (4), was divided to an appropriate number of intervals in order to have, during the motion, enough fingertips positions to give confident images about the spatial trajectories of these points. By connecting these positions, the complex surface bordering the active hand model workspace, was obtained. It is bordering the hand active workspace inside of which the assembly palm-fingers can move anywise. The complex surface could be used to verify the model correctness from the motion point of view, and to plan the hand motion by avoiding the collisions between its active workspace and obstacles in the neighborhood.

## Results

### Active space representation in MATLAB 7.0

**Figure 3:**
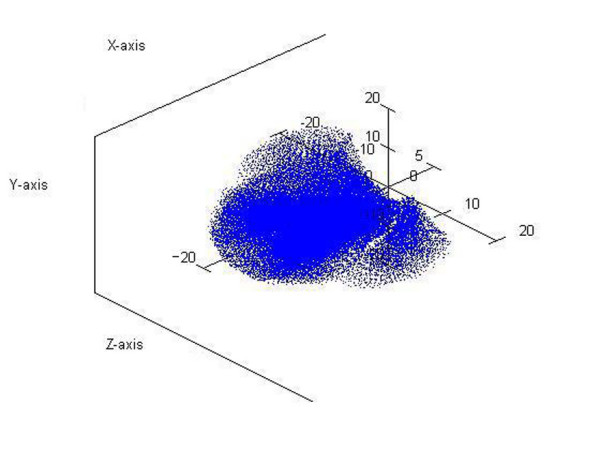
**Human hand's complex surface bordering the active workspace**. This complex surface was obtained when all articulations of the hand were moving simultaneously. The representation was realized in MATLAB 7.0 by using the transfer matrices for instantaneous values of joints variables *q *inside the domains defined by constraints. Any point on surface has its own coordinates smaller than *17 cm*, which represents the maximum length of the middle finger in static position.

If hand articulations are all simultaneously moving, the complex surface described by fingertips is represented in Figure 3 when every angular range was divided into two intervals. The representation was realized in MATLAB 7.0 by using the transfer matrices for instantaneous values of joints variables *q *inside the domains defined by constraints.

The program starts by reading the Denavit-Hartenberg parameters (theta max and min meaning joint variable *q *limits, alpha meaning bending angle, d meaning distance between two consecutive *Ox *axes and l meaning links length) of all fingers from external files in which they are defined.

temp=load('theta_min1');

theta_min1 = temp(:,1);

temp=load('theta_max1');

theta_max1 = temp(:,1);

temp=load('alpha1');

alpha1 = temp(:,1);

temp=load('l1');

len1 = temp(:,1);

temp=load('d1');

dist1 = temp(:,1);

After this step, it is asked from the user to give the number of intervals per angular range. The program will calculate the transfer matrix in these specific division points within the anatomical range given earlier in the external files (*theta max *and *theta min*), outputting a step variable for every joint.

level=input('Number of steps per joint level(>= 2): ');

Using this variable step the general matrix is calculated for the fingertips, by using several numbers of loops in parallel (number of joint levels per finger).

step1(1)=(theta_max1(1)-theta_min1(1))/level;

step1(2)=(theta_max1(2)-theta_min1(2))/level;

step1(3)=(theta_max1(3)-theta_min1(3))/level;

step1(4)=(theta_max1(4)-theta_min1(4))/level;

step1(5)=(theta_max1(5)-theta_min1(5))/level;

step1(6)=(theta_max1(6)-theta_min1(6))/level;

Within each loop a temporary transfer matrix is calculated, that in the next loop is used to calculate the farther one in the chain representing the general matrix. This is an example of calculus for the second phalanx for all fingers (the calculus is done in parallel for each finger).

for j1 = 1:level

. . .

for j6 = 1:level

T61=rotz(j61)*tra(len1(6),dist1(6))*rotx(alpha1(6));

. . .

   G61=G51*T61;

. . .

   j61=j61+step1(6);

. . .

In the innermost loop the program has all the values to calculate the general matrix for each finger.

for j7 = 1:level

T61=rotz(j61)*tra(len1(6),-dist1(6))*rotx(alpha1(6));

   . . .

      G61=G51*T61;

      . . .

         j61=j61+step1(6);

         . . .

            pozx(ink)=G61(1,4);

            pozy(ink)=G61(2,4);

            pozz(ink)=G61(3,4);

               ink=ink+1;

         . . .

      end

The results (concretized as parametrical equations) are stored in separate variables (pozx, pozy, pozz) which after the end of the iterations are plotted by using plot3 function. The points are 3D coordinates of the fingertips, so they can be plotted in 3D space (workspace) or in 2D space (projections).

It can be seen that any point on surface has its own coordinates smaller than *17 cm*, which represents the maximum length of the middle finger in static position.

Therefore, a sphere having the center in the fixed point *O*_0 _and the radius of *17 cm *covers the represented complex surface.

### Fingertips trajectories

It is very interesting to study the motion of the hand model when the radiocarpal articulation joints move separately and the joint coordinates *q*_1_, *q*_2_, *q*_3 _are variable one after the other. By stopping all other joints motions by stiffening the fingers to the palm in the relative position in Figure 2 there can be studied only the main motions of the human hand model as an assembly: flexion-extension provided by the joint 1, pronation-supination provided by the joint 2 and abduction-adduction provided by the joint 3. The fingertips trajectories are curves situated on spheres whose center is always the radiocarpal articulation point (intersection of joints 1, 2 and 3 axes).

## Discussion

The authors propose a human hand model that represents a new solution compared with other ones in literature. By comparing our model with that described in [1], the kinematical chains modeling the fingers are different. Except the thumb, all fingers in the proposed model have four DoFs, in which the metacarpophalangeal articulation has two DoFs provided by two orthogonal revolute joints, and every interphalangeal articulation has one DoF, provided by a corresponding revolute joint. For these four fingers, the structure is the same, but links lengths and distances are different. The thumb was conceived with three DoFs, two of them belonging to metacarpophalangeal articulation and the third to interphalangeal one. The thumb's joints orientations assure its global motion opposite to the other fingers motions as in the real hand.

By comparing to the model in [4], ours includes the palm as a rigid body that was articulated through the wrist to the forearm anatomical structure. Each finger is articulated to the palm by a two DoFs articulation. The wrist is considered a spherical joint modeled by superposing three orthogonal revolute joints, this assumption reflecting better the real situation. In our model in the wrist three reference frames are superposed, the first of them is the global (fixed) one *x*_0_*O*_0_*y*_0_*z*_0_. Each finger is separately moving conformably to its kinematical chain and the fingertips will describe their own trajectories with respect to the fixed reference frame.

Comparing to model from [7], we propose a hand model also based on the anatomical structure, but with a smaller number of DoFs, able to perform the motion similarly to the real hand. Its correctness was verified by studying the fingertips trajectories in the global reference frame planes.

The workspace is very important when visualize the progress of the hand after a surgical procedure, when during the recovery the workspace of the patient's hand is limited. In [9] was presented such a study for the wrist area, but they are using a different method to generate the workspace and reduce the hand to a point of interest placed on the thumb's tip. The workspace is, also, very important in any motion-planning problem through a space with objects, in order to avoid the interaction between the artificial hand and the objects which are not intended to be touched or grabbed. So, any kind of motion the proposed hand model will make will be for sure into the determined workspace (due to the motion constraints, based on which the complex surface from Figure 3 was determined). It will be easier to observe that the workspace is very similar with the one of human hand, if some simplified workspaces will be generated.

**Figure 4:**
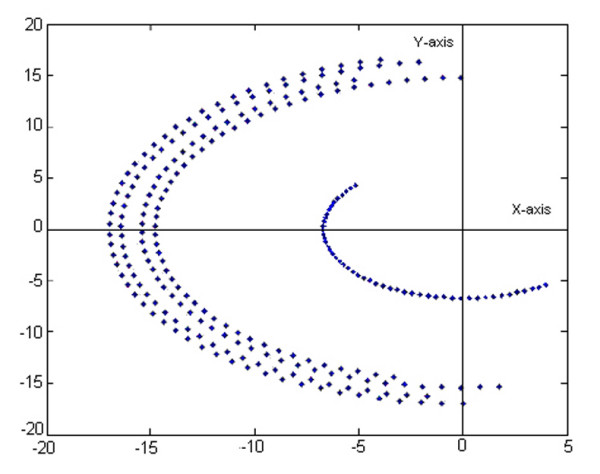
**Projection of the hand model active workspace in the plane orthogonal on the flexion-extension motion axis when the assembly palm_fingers is stiffened**. This space corresponds to the flexion-extension motion of the assembly palm-fingers (meaning only the joint 1 is moving). It can be observed that the active areas are asymmetrical disposed with respect to the horizontal axis. By stiffening all fingers' articulations, the assembly palm-fingers has a rigid body behavior and the fingers' tips trajectories are circle arcs situated in various planes parallels with *x*_0_*O*_0_*y*_0_. The thumb's trajectory is distinct placed with respect to the other four fingers whose trajectories are parallel. The middle finger describes a circle whose radius value is *17 cm*.

If only the joint 1 is moving, meaning rotation around the *O*_0_*z*_0 _axis, the corresponding active space is simplified comparing to that described in Figure 3. This space corresponds to the flexion-extension motion of the assembly palm-moving fingers. The study reveals that the active areas are asymmetrical disposed with respect to the horizontal *O*_0_*x*_0 _axis. By stopping all fingers articulations, the assembly palm-fingers has a rigid body behavior and the fingers' tips trajectories are circle arcs situated in various planes parallels with *x*_0_*O*_0_*y*_0 _(Figure 4). The thumb's trajectory is distinct placed with respect to the other four fingers whose trajectories are parallel. It is clearly observed that the middle finger (perfectly flat) describes a circle whose radius value is *17 cm*.

**Figure 5:**
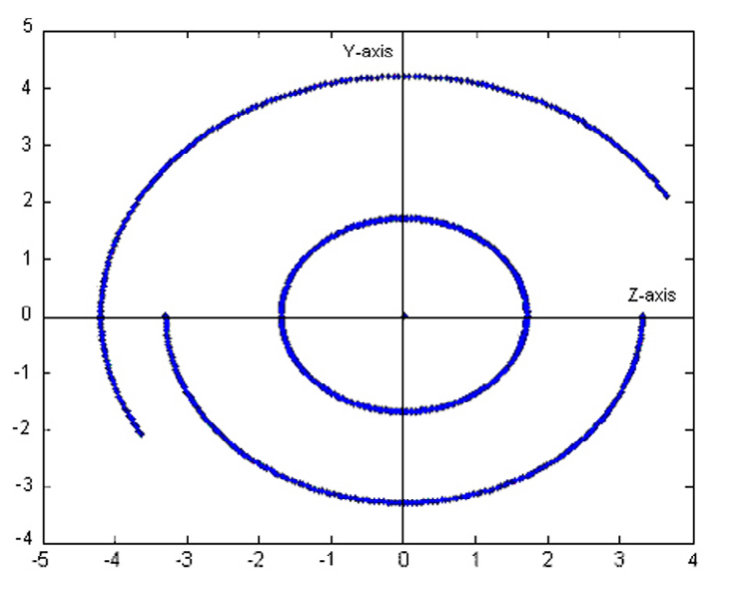
**Projection of the hand model active workspace in the plane orthogonal on the pronation-supination motion axis when the assembly palm-fingers is stiffened**. This complex surface covers the spaces described by the fingertips in pronation-supination motion (only joint 2 is moving). During this motion the thumb is always placed in 3D regions with coordinate *z *< 0. The fingers' tips trajectories are circle arcs situated in various planes parallels with *y*_0_*O*_0_*z*_0_. In the point whose coordinates are *y *= 0 *z *= 0, the middle finger is always pointed, because the rotation axis is even its static position. At 4 *cm *far from this point the circle described by the thumb is placed. At 2 *cm *from the origin, there are placed, symmetrically with respect to *O*_0_*y*_0_, the trajectories of index and ring finger. The pinkie's trajectory is the circle with radius of 3.3 *cm*.

If only the joint 2 is moving, meaning rotation around the *O*_0_*x*_0 _axis, the corresponding complex surface covers the spaces described by the fingers' tips in pronation-supination motion. During this motion the thumb is always placed in 3D regions with coordinate *z *< 0. By stopping all fingers articulations in this case, the fingers' tips trajectories are circle arcs situated in various planes parallels with *y*_0_*O*_0_*z*_0 _(Figure 5). In the point whose coordinates are *y *= 0 *z *= 0, the middle finger is always pointed, because the rotation axis is even its static position. At 4 *cm *far from this point the circle described by the thumb is placed. At 2 *cm *from the origin, there are placed, symmetrically with respect to *O*_0_*y*_0_, the trajectories of index and ring finger. The pinky's trajectory is the circle with radius of 3.3 *cm*.

**Figure 6:**
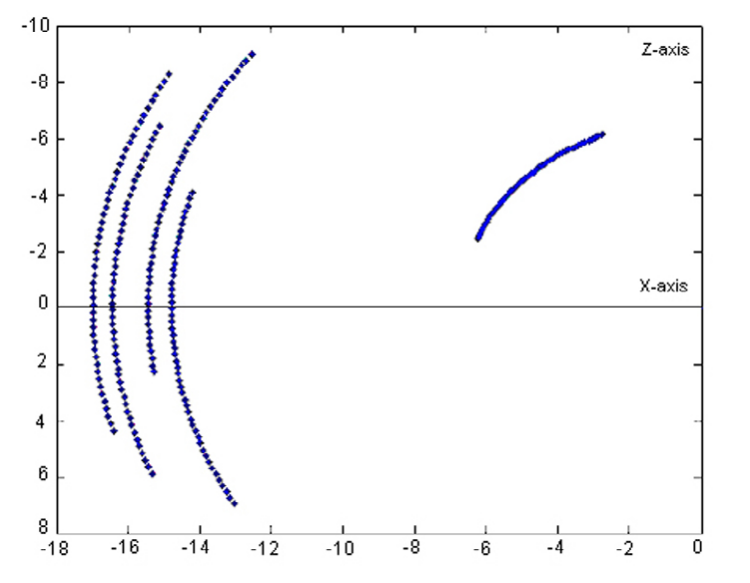
**Projection of the hand model active workspace in the plane orthogonal on the abduction-adduction motion axis when the assembly palm-fingers is stiffened**. The complex surface covering a simplified active workspace is described by the fingers' tips in abduction-adduction motion (only joint 3 is moving). All fingers' configurations are placed in the *x *< 0 regions, normal for the case when the palm is moving in the horizontal plane and the phalanges could be only below this one. The curves described by the fingertips during abduction-adduction, are circle arcs in planes parallels with *x*_0_*O*_0_*z*_0_. There can be observed the independent trajectory of the thumb and the group of trajectories of the other fingers.

If only the joint 3 is moving, meaning rotation around the *O*_0_*y*_0 _axis, the complex surface covering a simplified active workspace is described by the fingers' tips in abduction-adduction motion. All fingers' configurations are placed in the *x *< 0 regions, that situation satisfying the real execution of the motion when the palm is moving in the horizontal *x*_0_*O*_0_*z*_0 _plane and the phalanges could be only below this one. By considering the assembly palm-fingers as a rigid body, the curves described by the fingers' tips during abduction-adduction, are circle arcs in planes parallels with *x*_0_*O*_0_*z*_0 _(Figure 6). There are observed the independent trajectory of the thumb, as well as, the group of trajectories of the other fingers. Between them, the middle finger describes the circle arc of *17 cm *radius value.

The curves in Figure 4, 5, 6 were realized by dividing the joint variables domains into 5 intervals. If the number of intervals increases, the asked computing resources are very important and the quality of supplied information does not legitimate this action. The experience shows that 3–5 intervals are sufficiently good to obtain reliable results.

If the model is created with accuracy by respecting the type of motion provided by each articulation and the dimensions of articulated bones, it can function as the real organ providing the same motions.

**Figure 7:**
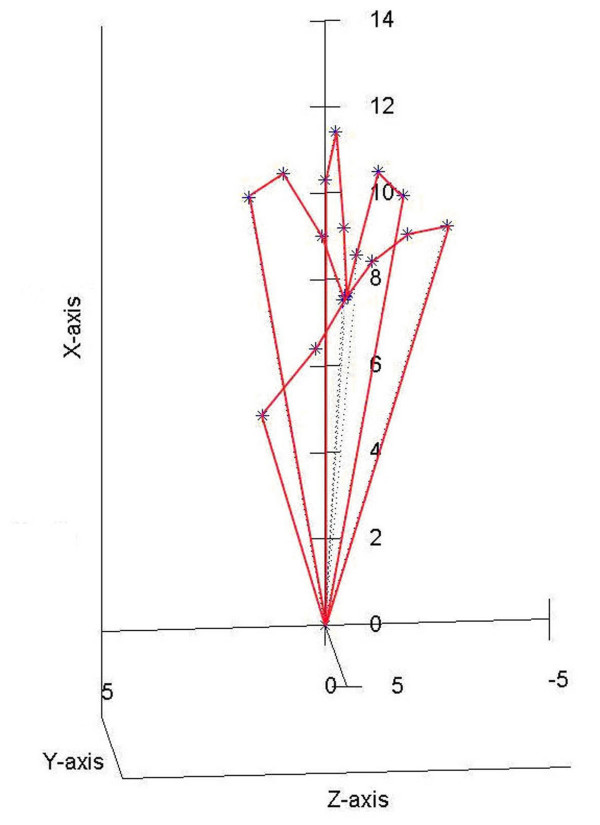
**Hand model with the fingertips closed together**. The model is capable to make fine movement as bringing all the fingertips together. It can be seen that the fingertips' positions are natural like and it can be obtained for joints variables values inside the limits imposed by anatomical constraints. As normal, the thumb stands in opposition with the other fingers.

**Figure 8:**
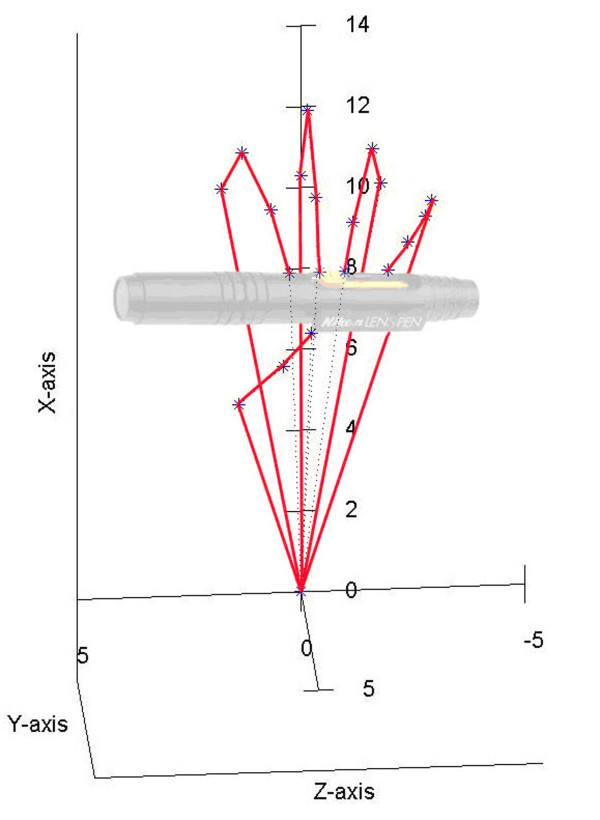
**Hand model sustaining a pen**. A ball point pen is turned between the fingers around its longitudinal axis. All the fingers are situated on the same side of the ball point pen, except the thumb which assures the object's stability during the motion. The object is stable and the fingers' positions are natural like.

**Figure 9:**
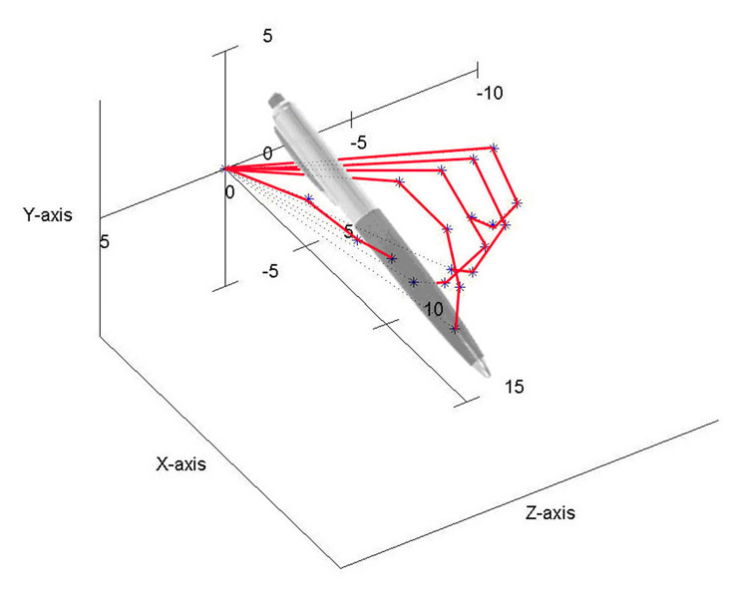
**Hand model in a writing position**. The model is capable to assure a writing position. The pinky, ring and middle fingers have the natural positions to maintain the hand stability during the writing. The middle finger sustains the ball point pen which is set between index and thumb.

The proposed model is capable to make special movements like power grip and dexterous manipulations. During them, the fingertips do not exceed the active workspace bordering by the surfaces represented in Figure 3. As examples, supporting this assertion, in Figure 7 it can be seen that the fingers have been moved in order to bring together the fingertips (position I). The fingertips position is a natural one having the average coordinates *x *= 8.860 *y *= 4.704 *z *= -0.034 *cm *and it can be obtained for joints variables values inside the limits imposed by anatomical constraints. The thumb moves in opposition with the other fingers in order to sustain the object, as it can be better observed in Figure 8. A ball point pen is turned between the fingers around its longitudinal axis. All the fingers are situated on the same side of the ball point pen, except the thumb which assures the object's stability during the motion (position II). Moving different phalanges a writing position of the hand model can be achieved (Figure 9). The pinky, ring and middle fingers are positioned in order to assure the hand stability during the motion due to the writing. In the same time, the middle finger sustains the ball point pen which is set between index and thumb (position III). The fingertip's coordinates (all of them inside the active workspace) and the values of joints variables assuring the mentioned positions are presented in Table 2.

**Table 2:** Fingertips' coordinates and joints variables in particular positions of the model

***Pos***.	***Finger***	***Fingertips coordinates ***[cm]			***Joints variables ***[^0^]						
		
		***X***	***y***	***z***	***q***_*1*_	***q***_*2*_	***q***_*3*_	***q***_*4*_	***q***_*5*_	***q***_*6*_	***q***_*7*_
***I***	1	4.69	8.63	0.01	80.00	0.00	0.00	15.00	35.00	10.00	-
	2	4.67	8.86	0.06	80.00	0.00	0.00	10.00	62.00	60.00	30.00
	3	4.66	8.97	0.00	80.00	0.00	0.00	0.00	53.00	80.00	30.00
	4	4.82	8.90	-0.17	80.00	0.00	0.00	-10.00	62.00	60.00	40.00
	5	4.68	8.94	-0.07	80.00	0.00	0.00	-15.00	80.00	20.00	35.00
***II***	1	4.45	7.24	-0.19	80.00	0.00	0.00	15.00	61.00	10.00	-
	2	3.94	8.12	1.77	80.00	0.00	0.00	5.00	65.00	75.00	25.00
	3	3.85	8.13	-0.00	80.00	0.00	0.00	0.00	60.00	90.00	30.00
	4	3.75	8.12	-1.77	80.00	0.00	0.00	-5.00	66.00	75.00	45.00
	5	2.55	8.12	-3.42	80.00	0.00	0.00	-7.00	63.00	80.00	45.00
***III***	1	9.69	2.38	-0.43	15.00	30.00	10.00	0.00	15.00	10.00	-
	2	12.68	1.14	-1.03	15.00	30.00	10.00	0.00	40.00	45.00	25.00
	3	8.80	0.19	-1.85	15.00	30.00	10.00	0.00	60.00	90.00	30.00
	4	8.48	0.18	-3.56	15.00	30.00	10.00	0.00	65.00	75.00	45.00
	5	7.15	0.48	-5.20	15.00	30.00	10.00	0.00	70.00	80.00	45.00

The table contains the fingertips' coordinates and the values of joints variables assuring the positions in Figures 7, 8, and 9. It can be seen that all of them are inside the active workspace.

**Figure 10:**
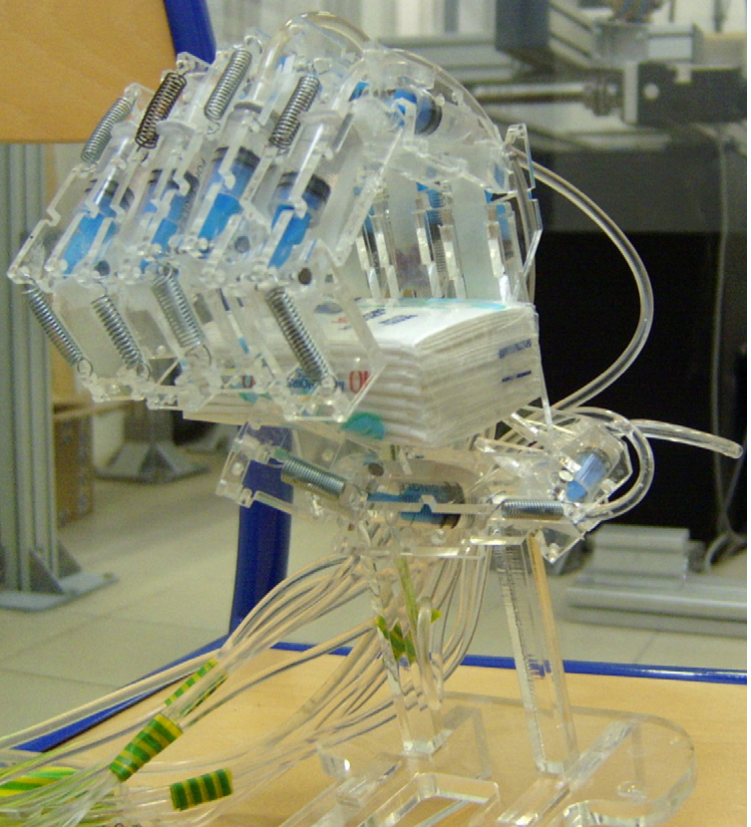
**The realized artificial hand**. The prosthesis has a hydraulic actuation, with the motors and pumps placed elsewhere (on the forearm). The structure of fingers respects the natural model, except the thumb which has only two phalanges. Although the model has a reduced number of DoFs it is capable to grab various objects.

In the same manner, reducing some degrees of freedom considered unimportant for a certain task, one can decide from the determined workspace if the task can be realized in this case or not. Based on this observation, we developed a hydraulically actuated human hand prosthesis model [26,27]. Because it was not yet feasible for us, technologically speaking, to design human hand prosthesis capable to realize all the natural functions, we considered that we should focus only on the prehension function. Having this in mind, we designed and constructed (Figure 10) a model very similar with the one from Figure 1. The difference consists in a reduced number of DoFs for the constructed model (no DoFs for adduction/abduction motion). We compensate this by placing the fingers in such way on the palm that objects having various shapes can be grabbed. A hydraulic actuation is used in order to place the motors and pump on the forearm to obtain a lighter prosthesis. We designed a special type of hydraulic actuation for flexing the fingers, having in mind to obtain a better linearity between the variation of the force exerted by the fingers and the actuating force (represented by the pressure of the hydraulic fluid.) The extension of the hand is made with repellent springs. There is lot of work to do in order to obtain a fully functional prosthesis. Still, the existing model is capable to grab objects of different sizes and shapes whit a reduced number of DoFs.

## Conclusion

The first step to create a functional and adjustable human hand prosthesis is to model the architecture of the natural system, as well as its motion functions. If the model of the human hand is created with accuracy by respecting the type of motion provided by each articulation and the dimensions of articulated bones, it can function as the real organ providing the same motions. The main problem is that the human hand is hard to model due to its kinematical chains submitted to motion constraints. If the application does not impose a fine manipulation it is not necessary to create a model as complex as the human hand is. But always, the hand model has to perform a certain lot of motions in imposed workspace architecture whichever should be the practical application. The proposed kinematical model can help to choose which model joints could be eliminated in order to preserve only the motions important for that kind of application. The study shows that all models, simplified or not, exhibit a pronounced similitude with the real hand motion, validated by the fingertips' computed trajectories.

## Competing interests

The author(s) declare that they have no competing interests.

## Authors' contributions

DD created the model based on the real human hand, computed the kinematical equations of the model and interpreted the results on the fingertips trajectories by comparing them with the real situations. VP and MD participated with suggestions on the proposed model in order to correspond to a functional hand prosthesis design. LU participated in the design of the study, carried out the background and the study of the natural model and drafted the manuscript. KM realized the MATLAB programming and helped to draft the manuscript. All authors read and approved the final manuscript.
